# Identification of critical sites for glycine- and cesium-dependent activation of the RNA-edited glycine receptor α3L

**DOI:** 10.3389/fnmol.2026.1918246

**Published:** 2026-10-09

**Authors:** Yulia Novykova, Steffen Fricke, Jochen C. Meier

**Affiliations:** Division Cell Physiology, Institute for Cell- and Neurobiology, Technical University Braunschweig, Braunschweig, Germany

**Keywords:** agonist binding site, cesium, glycine, glycine receptor (GlyR), receptor activation

## Abstract

Glycine receptors (GlyRs) are chloride permeable ligand-gated ion channels that play an important role in synaptic transmission in the central nervous system. Besides glycine as neurotransmitter, cesium (Cs^+^) was shown to activate homomeric GlyRs with α2 and α3 subunits, and a recent study revealed the position D194 as essential for activation of RNA-edited GlyR α3^185L^ at the cell surface. This position in the N-terminal extracellular ligand-binding domain belongs to a series of amino acid positions (D141, E192, D194 and D91, D97, E110, D114, Y161) predicted to be involved in Cs^+^ binding. In the present study, we mutated the negatively charged amino acids D91, D97, E110, and D114 as well as the neutral polar amino acid Y161 to code for alanine with a neutral hydrophobic side chain or lysine with a positively charged side chain and performed patch-clamp analysis as well as surface GlyR staining using transfected HEK293T cells. We found that substitutions D97A/K and Y161K resulted in a complete loss of receptor function in response to glycine or Cs^+^, whereas mutations at D91 and E110 retained detectable current responses, with E110A/K mainly affecting glycine-evoked currents. Y161A retained at least some Cs^+^-dependent GlyR responsiveness at concentrations above 5 mM, indicating that a positive charge (K) at position Y161 is detrimental to glycine-dependent GlyR activation. However, as no mutant was identified that responded to glycine while lacking Cs^+^-evoked activity, these findings suggest that Cs^+^-mediated GlyR activation involves the same structural framework required for glycine-induced channel opening.

## Introduction

Glycine receptors (GlyRs) are ligand-gated chloride channels belonging to the family of pentameric Cys-loop receptors and mediate a major component of fast inhibitory neurotransmission in the central nervous system (CNS). RNA splicing generates short or long variants of the glycine receptor α3 (GlyR α3) that differ within the intracellular loop between transmembrane domains (TM) 3 and 4 by inclusion of exon 8A of the *Glra3* gene coding for the long GlyR α3L variant. Although this region does not directly participate in ligand binding or ion conduction, splice-dependent differences can influence receptor regulation and trafficking (Nikolic et al., 1998; Meier et al., 2016). RNA editing represents a less frequent but functionally important post-transcriptional modification. In glycine receptor transcripts, cytidine-to-uridine (C-to-U) RNA editing results in a proline-to-leucine substitution within the extracellular domain at positions P185 and P192 of α2- and α3-GlyRs, respectively (Legendre et al., 2009). This modification increases glycine sensitivity and is therefore associated with a gain-of-function phenotype (Meier et al., 2005; Meier et al., 2016). Elevated levels of *Glra3*-encoded GlyR α3 mRNA have been observed in temporal lobe epilepsy, suggesting that RNA editing-dependent modulation of GlyR function may contribute to pathological changes in neuronal network homeostasis, resulting in cognitive deficits or anxiety, depending on the neuronal cell type that is concerned (Winkelmann et al., 2014; Çaliskan et al., 2016; Meier et al., 2016). In this context, alternative splicing and RNA editing contribute to the structural and functional diversity of α3-GlyRs.

In addition to classical neurotransmitters and pharmacological ligands, GlyRs can be influenced by certain monovalent cations. While Na^+^, K^+^, and Li^+^ ions do not induce measurable GlyR activation, Cs^+^ has been shown to activate at least α2- and α3-GlyRs. In fact, recent studies demonstrated that Cs^+^ and also NH_4_^+^ can act as agonists of α2- and α3-GlyRs, with their RNA-edited GlyR variants exhibiting increased responsiveness to both, Cs^+^ and NH_4_^+^ compared to unedited receptors at millimolar concentrations (Kankowski et al., 2018; Fricke et al., 2023). In 2025, we characterized the predicted allosteric Cs^+^ binding site involving amino acid positions D141, E192, and D194 in close vicinity of the editable P185 site in the F-loop region and identified D194 as essential for activation of α3L-GlyRs at the cell surface and E192 as relevant for desensitization of α3L-GlyR current responses (Harnau et al., 2025).

In the present study, we analysed D91, D97, E110, D114, and Y161 in or near the orthosteric glycine binding site of the RNA-edited GlyR α3L^185L^ (Figure 1A) and performed whole-cell patch-clamp experiments using transfected HEK293T cells.

**Figure 1:**
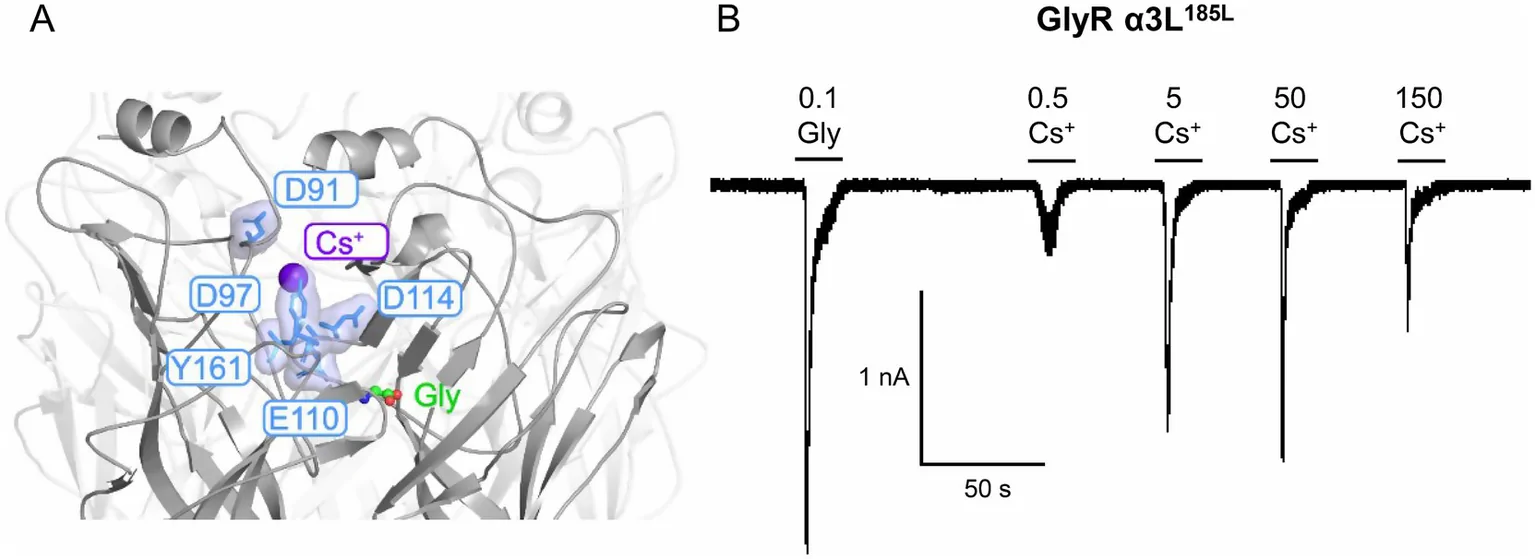
Structure-function relationships of glycine- and Cs^+^-dependent GlyR activation. **(A)** Prediction of Cs^+^ binding site overlapping with the glycine binding site, comprising positions D91, D97, E110, D114, and Y161, as previously postulated (Fricke et al., 2023). **(B)** Representative patch clamp recording from HEK293T cells expressing the non-mutated GlyR α3L^185L^ subjected to 0.1 mM glycine or various Cs^+^ concentrations (in mM).

We found that substitutions at D97A/K (meaning D97A or D97K; “/” used throughout if alanine or lysine substitutions are meant) and Y161K resulted in a pronounced loss of receptor function in response to glycine or Cs^+^, whereas mutations at D91, E110, and D114 retained detectable current responses with the glycine concentration of 100 µM that is subsaturating for GlyR α3L^185L^. In particular, D91A/K variants affected both, glycine- and Cs^+^-evoked current responses, while E110A/K variants primarily reduced glycine-evoked current responses. Determination of the apparent affinities (EC_50_) in response to 0.1, 1.0, 5, 10, 50, 100, and 500 µM glycine of GlyR α3L^185L^ and its mutants D91A/K, E110A/K, and D114A/K also revealed that mostly alanine mutants displayed increased EC_50_ values, indicating reduced apparent affinities. Accordingly, the Hill slopes were reduced in most of these cases, suggesting reduced cooperativity of ligand binding. Furthermore, Y161A and Y161K substitutions almost completely abolished glycine-dependent GlyR activation, while Y161A retained at least some Cs^+^-dependent GlyR responsiveness at concentrations above 5 mM, indicating that a positive charge at position Y161 is detrimental to glycine-dependent GlyR activation. Thus, Cs^+^ was able to activate several mutated α3L-GlyR variants studied here, and at higher concentrations (50 mM) Cs^+^-evoked currents were comparable to or even exceeded those induced by glycine. However, as no mutant was identified that responded to glycine while lacking Cs^+^-evoked activity, these findings suggest that Cs^+^-mediated GlyR activation involves the same structural framework required for glycine-induced channel opening and does not represent an independent receptor activation mechanism. Alternatively, there might be other positions than the ones studied by us here and previously (Harnau et al., 2025) that could be specifically involved in Cs^+^-dependent GlyR α3L^185L^ activation. However, the results unexpectedly unmask D91, E110, and D114 as critical positions for glycine-dependent GlyR α3L^185L^ activation, and they identify D97 as essential for GlyR α3L^185L^ activation in general.

## Material and methods

### Molecular constructs

The *Glra3*-encoded GlyR α3L (NCBI accession number NM_080438.4) P185L-expressing construct was mutated by PCR using primers with the corresponding mutations leading to D91A/K, D97A/K, E110A/K, D114A/K, and Y161A/K substitutions. For analysis of GlyR surface expression the HA-epitope YPYDVPDYA was inserted likewise between the second and third amino acid position in the mature signal peptide-cleaved GlyR α3, which is a location suitable for GlyR surface staining using epitope tags (Eichler et al., 2009; Förstera et al., 2014). Ligation was carried out using the NEBuilder Hifi DNA Assembly Master Mix (New England Biolabs, #E2621). Transformation into chemically competent *Escherichia coli* Xl1blue by heat shock and subsequent isolation and purification of plasmids with Monarch Spin Plasmid Miniprep Kit (New England Biolabs, #T1110L) and PureLink^TM^ HiPure Plasmid Midiprep Kit (Invitrogen^TM^, #K210005) led to the constructs used in this study. Primer synthesis and DNA sequencing were carried out by Eurofins Genomics Germany GmbH. The GlyR transcription was driven by a CMV promotor and EGFP was co-expressed via an IRES.

### HEK293T culture and gene expression

Human embryonic kidney (HEK) 293T cells (DSMZ, ACC 635) were cultured in a humidified incubator at 37 °C and 5% CO_2_ in T25 flasks containing 5 mL DMEM (Gibco^TM^, #41965-062) with 10% fetal calf serum (Gibco^TM^, #10500064) and 1% penicillin/streptomycin (Gibco^TM^, #15140122). Cells were passaged twice a week and plated onto 35 mm culture dishes to reach about 800,000 cells for the subsequent transfection with FuGENE HD transfection reagent (Promega, #E2311). The transfection was carried out following the protocol of the manufacturer using 1 µg of plasmid DNA. After incubation over-night, the cells were enzymatically detached and seeded on 13 mm glass coverslips (VWR, #631-1578). Prior to seeding, the coverslips were pre-treated with 10 M NaOH for 3 h at 100 °C, sterilized at 180 °C for 8 h and coated with 0.5 mg/mL poly-L-lysine hydrobromide (Sigma Aldrich, #P2636) for 3 h at 37 °C. After 1–2 h sufficient attachment of the cells to the coverslip surface was achieved and whole-cell patch clamp experiments were performed. For GlyR surface staining, 10,000 HEK293T cells were seeded on lysine-coated coverslips in 24 well plates. Transfection was performed on the next day with FuGENE and 0.25 µg plasmid.

### Electrophysiology

The coverslips with the transfected HEK293T cells were placed in an open imaging chamber under a Nikon Ts2R microscope, and whole-cell voltage patch clamp was performed using an EPC 10 amplifier and Patchmaster software (HEKA). GlyR-expressing cells were identified according to EGFP fluorescence detection using a pE-4000 illumination system (CoolLED) and a Nikon sCMOS pco.edge camera. Borosilicate glass capillaries (World Precision Instrument, #1B150F-4) were pulled with a PC10 puller (Narishige) and their resistances lay between 4 and 8 MΩ when filled with the following intracellular solution (in mM): CsCl (130), NaCl (5), CaCl_2_ (0.5), MgCl_2_ (1), EGTA (5), and HEPES (30); pH = 7.2 (CsOH). We used Cs^+^ as a substitute for potassium ions in the internal solution as described before (Fricke et al., 2023; Harnau et al., 2025) to block voltage-gated potassium channels (Clay and Shlesinger, 1984), which improves space-clamp control and does not confound the interpretation of extracellular Cs^+^-induced receptor activation. The extracellular solution (ES) contained (in mM): NaCl (140), KCl (5), MgCl_2_ (1), CaCl_2_ (2), HEPES (10), and glucose (10); pH = 7.4 (NaOH). The solutions containing various concentrations of Cs^+^ (0.5 mM, 5 mM and 50 mM) were prepared by mixing ES with the corresponding amount of 150 mM Cs^+^ solution (in mM): CsCl (0.5, 5, or 50), MgCl_2_ (1), CaCl_2_ (2), HEPES (10), and glucose (10); pH 7.4 (CsOH). To ensure rapid fluid exchange a gravity-driven perfusion system equipped with a perfusion pencil with a 360 µm tip (AutoMate Scientific, #04-08-250) and a suction pump were utilized. ES was applied while opening the HEK293T cells and in between the various CS^+^ solutions. The voltage clamp holding potential was −50 mV and the series resistances (R_s_) were monitored by 50 ms long −5 mV voltage pulses every 5 s. The patch data were recorded at 20 kHz and filtered with a 2.8 kHz Bessel filter. All experiments were carried out at room temperature.

### GlyR surface staining and confocal microscopy

For the detection of HA-tagged α3L-GlyRs at the cell surface, the transfected HEK293T cells were washed with 0.1% PBS-gelatin and then incubated with 50 µL primary antibody (chicken anti-HA, 1:200) for 15 min at 37 °C in the incubator. Following two wash steps with PBS-gelatin the cells were fixed with 4% PFA/sucrose solution for 15 min. Following three further wash steps the coverslips were treated with 50 µL secondary antibody (anti-chicken IgY-Cy3, 1:200) for 45 min at room temperature. Finally, the coverslips with stained cells were washed twice with PBS-gelatin, three times with PBS, and mounted with Vectashield containing DAPI and sealed with nail polish. Fluorescence imaging was performed using a Stellaris 5 confocal laser scanning microscope by Leica Microsystems. Images were recorded in 8-bit with 1,024 × 1,024 resolution and fluorescence was excited at 552 nm for Cy3, 489 nm for EGFP and 405 nm for DAPI. Emission was recorded in the interval of 557–650 nm for Cy3, 494–550 nm for EGFP and 420–460 nm for DAPI, respectively. Laser intensity (DAPI: 2%, EGFP: 2.5%, Cy3: 4.5%) and gain settings (DAPI: 30%, EGFP: 30%, Cy3: 45%) were kept constant and recording was performed with 2x average to reduce background fluorescence.

### Data analysis and statistics

Electrophysiology data analysis was performed offline with IGOR Pro software and a custom written tool by M. Semtner (Raltschev et al., 2016). Peak amplitudes were measured, baseline subtracted, and Cs^+^-evoked currents were normalized to the maximum current amplitudes in response to 0.1 mM glycine. Cells with series resistance (R_s_) higher than 35 MΩ were excluded. Statistical analysis was performed using the GraphPad Prism software. The absolute and normalized amplitudes were checked for normal distribution using the Kolmorogov-Smirnov test and examined by repeated-measures two-way ANOVA test with the recommended Geisser-Greenhouse correction and Tukey´s multiple comparison test with individual variances computed for each comparison in Prism 9 (GraphPad). EC_50_ and Hill slope values were determined from glycine (0.1, 1, 5, 10, 50, 100, and 500 µM) and Cs^+^ (0.5, 5, 50, and 150 mM) concentration-response curves using nonlinear regression with a variable-slope model in GraphPad Prism 9 (GraphPad). Fluorescence images of surface-GlyRs were analyzed by comparison of the whole image Cy3 fluorescence in ratio to the number of transfected cells. Background fluorescence in the Cy3 channel was determined using ImageJ by measuring unspecific fluorescence observed in the negative control, i.e., the HEK293T cells expressing EGFP only, and subsequently subtracted. Finally, the data were normalized to the results of the non-mutated GlyR α3L^185L^. Statistical analyses were performed in Prism 9 (GraphPad) using the Kruskal-Wallis test with Dunn´s multiple comparison test as the fluorescence ratios were not normally distributed as determined with Kolgomorov-Smirnov test.

## Results

Receptor activation was examined in HEK293T cells by first applying 100 µM glycine, a subsaturating condition of GlyR activation with GlyR α3L^185L^ being closer to saturation than GlyR α3L^185P^ (Meier et al., 2005; Legendre et al., 2009; Fricke et al., 2023; Harnau et al., 2025), followed by successive applications of Cs^+^ (0.5, 5, 50, and 150 mM) at a holding potential of −50 mV. GlyR α3L^185L^ served as the non-mutated reference, as no mutations within the proposed Cs^+^ binding region near the glycine binding pocket (Figure 1A) were present. Figure 1B shows a representative trace obtained with this construct. As observed previously (Fricke et al., 2023; Harnau et al., 2025), cells expressing GlyR α3L^185L^ displayed typical Cs^+^-triggered currents at all concentrations, with an activation profile consisting of a rapid peak, desensitization to a steady-state level and a return to baseline upon washout. We also determined the apparent affinities (EC_50_) and Hill slopes for GlyR α3L^185L^ and its mutants D91A/K, E110A/K, and D114A/K.

The results shown in Figure 2 address the effect of the different mutations studied here on the glycine- and Cs^+^-induced current responses. Representative current traces and quantification of peak current responses of the non-mutated GlyR α3L^185L^ as well as alanine and lysine substitutions at the positions D91, D97, E110, D114, and Y161 are shown. Quantitative data are reported as absolute current responses and glycine-normalized ones (I_Cs_^+^/I_Gly_, Table 1).

**Table 1:** Maximum absolute (in nA) and normalized current amplitudes of HEK293T cells expressing the non-mutated or various mutated GlyR α3L^185L^.

		0.1 mM glycine	0.5 mM Cs^+^	5 mM Cs^+^	50 mM Cs^+^	150 mM Cs^+^
GlyR α3L^185L^ (*n* = 17)	Abs.	1.67 ± 0.26 (SD = 1.08)	0.16 ± 0.06 (SD = 0.24)	0.64 ± 0.13 (SD = 0.53)	1.79 ± 0.27 (SD = 1.10)	1.80 ± 0.28 (SD = 1.17)
	Norm.	1.00 ± 0.00 (SD = 0.00)	0.12 ± 0.03 (SD = 0.12)	0.41 ± 0.06 (SD = 0.26)	1.38 ± 0.30 (SD = 1.22)	1.31 ± 0.25 (SD = 1.05)
D91A (*n* = 11)	Abs.	0.50 ± 0.12 (SD = 0.40)	0.04 ± 0.01 (SD = 0.02)	0.15 ± 0.02 (SD = 0.06)	0.67 ± 0.09 (SD = 0.31)	0.84 ± 0.12 (SD = 0.40)
	Norm.	1.00 ± 0.00 (SD = 0.00)	0.16 ± 0.03 (SD = 0.11)	0.47 ± 0.10 (SD = 0.34)	1.92 ± 0.36 (SD = 1.18)	2.27 ± 0.39 (SD = 1.29)
D91K (*n* = 11)	Abs.	0.76 ± 0.12 (SD = 0.36)	0.05 ± 0.00 (SD = 0.01)	0.17 ± 0.03 (SD = 0.07)	0.79 ± 0.14 (SD = 0.47)	1.07 ± 0.16 (SD = 0.51)
	Norm.	1.00 ± 0.00 (SD = 0.00)	0.08 ± 0.01 (SD = 0.04)	0.24 ± 0.03 (SD = 0.08)	1.20 ± 0.23 (SD = 0.73)	1.64 ± 0.27 (SD = 0.86)
D97A (*n* = 7)	Abs.	0.05 ± 0.01 (SD = 0.03)	0.05 ± 0.01 (SD = 0.02)	0.06 ± 0.01 (SD = 0.02)	0.05 ± 0.01 (SD = 0.02)	0.05 ± 0.00 (SD = 0.01)
	Norm.	1.00 ± 0.00 (SD = 0.00)	1.08 ± 0.14 (SD = 0.38)	1.35 ± 0.19 (SD = 0.51)	1.13 ± 0.30 (SD = 0.80)	1.33 ± 0.39 (SD = 1.03)
D97K (*n* = 4)	Abs.	0.04 ± 0.01 (SD = 0.03)	0.03 ± 0.01 (SD = 0.02)	0.04 ± 0.01 (SD = 0.02)	0.04 ± 0.01 (SD = 0.01)	0.05 ± 0.01 (SD = 0.02)
	Norm.	1.00 ± 0.00 (SD = 0.00)	0.89 ± 0.16 (SD = 0.32)	1.43 ± 0.30 (SD = 0.60)	1.35 ± 0.32 (SD = 0.63)	2.03 ± 0.88 (SD = 1.76)
E110A (*n* = 12)	Abs.	0.35 ± 0.14 (SD = 0.48)	0.06 ± 0.01 (SD = 0.03)	0.32 ± 0.10 (SD = 0.35)	1.26 ± 0.27 (SD = 0.92)	1.24 ± 0.27 (SD = 0.95)
	Norm.	1.00 ± 0.00 (SD = 0.00)	0.75 ± 0.23 (SD = 0.79)	2.59 ± 0.70 (SD = 2.41)	12.90 ± 4.22 (SD = 14.61)	12.76 ± 4.27 (SD = 14.80)
E110K (*n* = 12)	Abs.	0.33 ± 0.08 (SD = 0.28)	0.10 ± 0.01 (SD = 0.05)	0.42 ± 0.08 (SD = 0.27)	1.05 ± 0.18 (SD = 0.61)	1.12 ± 0.15 (SD = 0.53)
	Norm.	1.00 ± 0.00 (SD = 0.00)	0.51 ± 0.14 (SD = 0.47)	2.57 ± 1.17 (SD = 4.06)	7.76 ± 3.47 (SD = 12.03)	7.01 ± 2.44 (SD = 8.46)
D114A (*n* = 10)	Abs.	0.92 ± 0.23 (SD = 0.74)	0.13 ± 0.05 (SD = 0.17)	0.31 ± 0.11 (SD = 0.34)	0.88 ± 0.23 (SD = 0.73)	0.97 ± 0.22 (SD = 0.68)
	Norm.	1.00 ± 0.00 (SD = 0.00)	0.15 ± 0.02 (SD = 0.08)	0.32 ± 0.04 (SD = 0.13)	1.08 ± 0.18 (SD = 0.58)	1.52 ± 0.41 (SD = 1.31)
D114K (*n* = 10)	Abs.	1.50 ± 0.23 (SD = 0.72)	0.17 ± 0.05 (SD = 0.14)	0.73 ± 0.11 (SD = 0.35)	1.53 ± 0.20 (SD = 0.63)	1.16 ± 0.15 (SD = 0.46)
	Norm.	1.00 ± 0.00 (SD = 0.00)	0.13 ± 0.03 (SD = 0.09)	0.50 ± 0.06 (SD = 0.19)	1.12 ± 0.13 (SD = 0.40)	0.86 ± 0.12 (SD = 0.38)
Y161A (*n* = 10)	Abs.	0.06 ± 0.03 (SD = 0.08)	0.03 ± 0.00 (SD = 0.01)	0.05 ± 0.01 (SD = 0.03)	0.24 ± 0.05 (SD = 0.17)	0.42 ± 0.12 (SD = 0.39)
	Norm.	1.00 ± 0.00 (SD = 0.00)	0.99 ± 0.20 (SD = 0.64)	1.46 ± 0.35 (SD = 1.09)	6.66 ± 1.50 (SD = 4.75)	10.32 ± 2.82 (SD = 8.91)
Y161K (*n* = 4)	Abs.	0.02 ± 0.00 (SD = 0.01)	0.02 ± 0.00 (SD = 0.01)	0.01 ± 0.00 (SD = 0.01)	0.02 ± 0.00 (SD = 0.00)	0.02 ± 0.00 (SD = 0.00)
	Norm.	1.00 ± 0.00 (SD = 0.00)	1.27 ± 0.18 (SD = 0.37)	1.00 ± 0.19 (SD = 0.38)	1.47 ± 0.28 (SD = 0.56)	1.26 ± 0.43 (SD = 0.86)

Data are listed as mean ± SEM (and SD).

**Figure 2:**
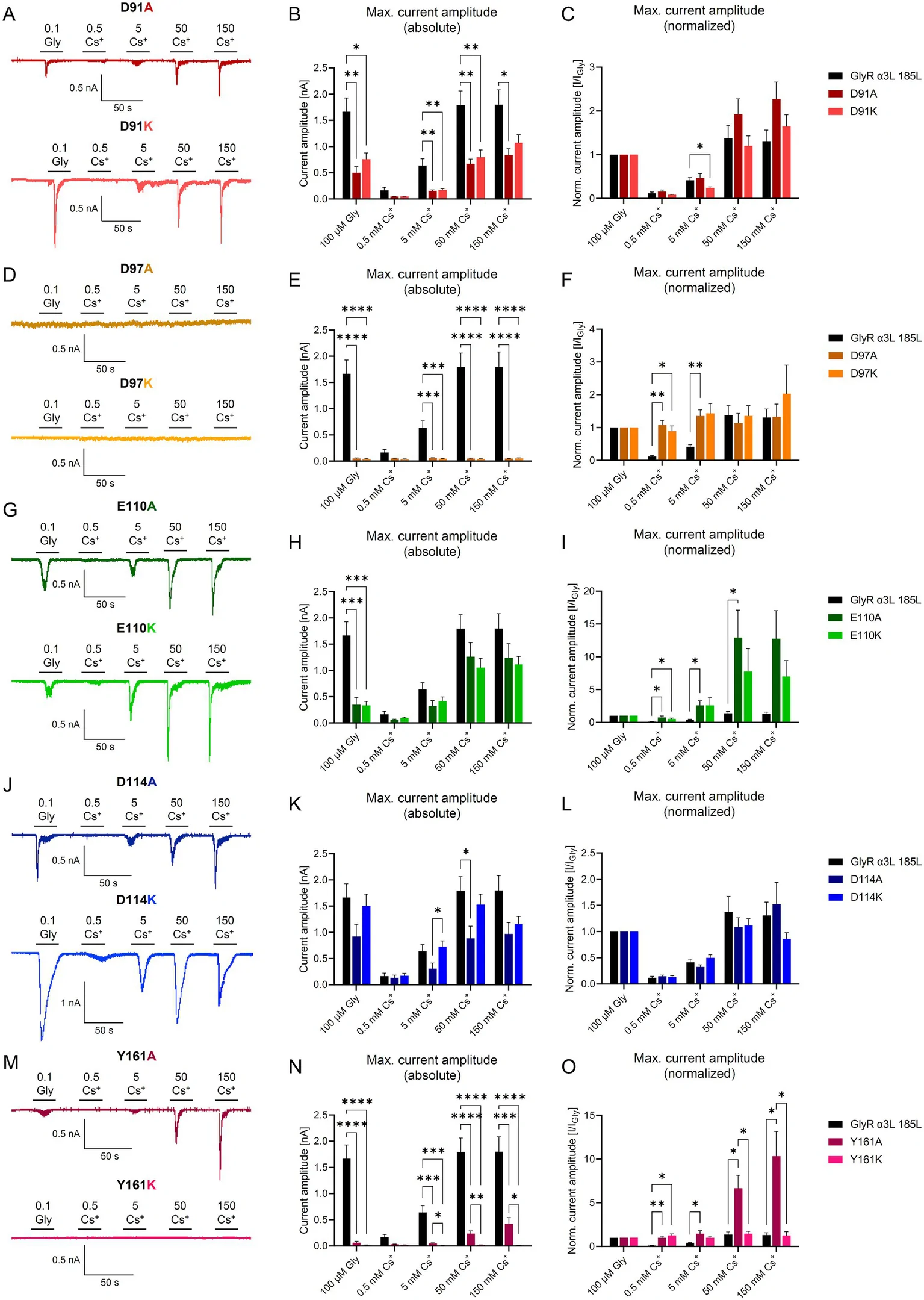
Effects of alanine and lysine substitutions at positions D91, D97, E110, D114, and Y161 on glycine- and Cs^+^-dependent GlyR current responses. **(A–O)** Representative whole-cell patch clamp recordings of HEK293T cells expressing non-mutated or mutated GlyR α3L^185L^ (left) as well as the corresponding absolute (center) and glycine-normalized current amplitudes (right) in response to 100 µM glycine and various Cs^+^ concentrations of GlyR α3L^185L^ (*n* = 17), D91A/K (**A–C**, *n* = 11 each), D97A/K (**D–F**, *n* = 7 and *n* = 4), E110A/K (**G–I**, *n* = 12 each), D114A/K (**J–L**, *n* = 10 each) and Y161A/K (**M–O**, *n* = 10 and *n* = 4). Data are presented as mean ± SEM (*p < 0.05, **p < 0.01, ***p < 0.001, ****p < 0.0001).

We first focused on the position D91 (Figures 2A–C). The current traces and quantification of the absolute current responses clearly shows that D91A/K substitutions resulted in significantly reduced currents in response to both, glycine and Cs^+^ (Figures 2A,B, Table 1, *p < 0.05, **p < 0.01). Accordingly, the normalized current responses were not significantly different, except at 5 mM Cs^+^ with the D91K variant reducing potency of this rather low Cs^+^ concentration (Figure 2C, Table 1, *p < 0.05). Furthermore, the apparent affinities for glycine and Cs^+^ (EC_50_ values, Table 2) are reduced and less cooperativity of ligand-gating (Hill slopes, Table 2) was observed. Thus, alanine and lysine substitutions at position D91 impair responses to both, glycine and Cs^+^.

**Table 2:** EC_50_ and Hill slopes determined with HEK293T cells expressing non-mutated and mutated GlyR α3L^185L^ for glycine- and Cs^+^-dependent current responses.

	EC_50_		Hill slope	
	Glycine (µM)	Cs^+^(mM)	Glycine	Cs^+^
GlyR α3L^185L^	8.60	6.85	1.92	2.84
D91A	13.29	23.47	1.94	1.21
D91K	18.12	32.00	1.22	1.13
E110A	15.41	6.62	1.47	4.56
E110K	7.59	8.45	2.17	1.41
D114A	32.01	12.74	1.09	1.41
D114K	7.01	5.04	2.36	ND

ND, not determinable due to unstable fitting.

While D91 mutations had moderate to strong effects on receptor activation in response to the agonists studied here, alanine and lysine substitutions at position D97 had detrimental effects on current responses (Figures 2D–F). The representative current traces in Figure 2D show absence of clear current peak responses. Likewise, quantification of the absolute current responses shows that D97A/K substitutions were not compatible with receptor activation in response to glycine or Cs^+^ (Figure 2E, Table 1, ***p < 0.001, ****p < 0.0001). The normalized data show some significant differences at low Cs^+^ concentrations (0.5 and 5 mM, Figure 2F, Table 1, *p < 0.05, **p < 0.01). However, as it is difficult to quantify peak current responses in the absence of a clearly identifiable peak, the normalized data should be interpreted with care. Furthermore, for these reasons, we were not able to determine EC_50_ values and Hill slopes.

We next investigated the effects of alanine and lysine substitutions at position E110 (Figures 2G–I). Quantification of the absolute current responses in revealed that E110A/K substitutions mainly affected glycine-evoked maximal current responses while Cs^+^-dependent receptor activation was not compromised (Figures 2G,H, Table 1, ***p < 0.001). Likewise, quantification of the normalized current responses revealed significant differences at the varying Cs^+^ concentrations (Figure 2I, Table 1, *p < 0.05). Determination of EC_50_ values and Hill slopes (Table 2) confirmed this trend but also revealed that the alanine substitution (E110A) reduced apparent glycine-affinity (increased EC_50_ value) and reduced cooperativity (decreased Hill slope). Thus, a neutral charge (alanine) at E110 appears to impair glycine-dependent GlyR α3L^185L^ activation, while Cs^+^-dependent responses were not affected.

The position D114 is just 4 amino acids downstream of E110 and was studied next (Figures 2J–L). While absolute maximal current amplitudes of D114 mutants in response to 100 µM glycine appeared to be unaltered compared to the non-mutated GlyR α3L^185L^, D114A showed reduced responses to 5 mM Cs^+^ compared to D114K as well as reduced currents in response to 50 mM Cs^+^ compared to the non-mutated receptor, with a slight but non-significant reduction in 100 µM glycine-evoked currents (Figures 2J,K, Table 1, *p < 0.05). When Cs^+^-evoked current amplitudes were normalized to the glycine response, no alterations between conditions were present (Figure 2L, Table 1). However, the apparent glycine- and Cs^+^-affinities of the D114A mutant were reduced and Hill slopes were also decreased (Table 2), indicating that a neutral charge (alanine) in this region impairs glycine- and Cs^+^-dependent GlyR α3L^185L^ activation.

Finally, we investigated effects of alanine and lysine substitutions at position Y161 (Figures 2M–O). Both substitutions at this position almost completely abolished glycine-evoked absolute current responses (Figures 2M,N, Table 1, ****p < 0.0001). While receptors with the positive charge of lysine at this position did not generate currents at all in response to both, glycine and Cs^+^, the neutral alanine preferentially affected glycine-dependent responses (Figures 2M,N, Table 1, *p < 0.05, **p < 0.01, ***p < 0.001, ****p < 0.0001). Likewise, the normalized data are in agreement with the observation that Cs^+^-evoked currents were still detectable with the Y161A variant (Figure 2O, Table 1, *p < 0.05, **p < 0.01).

Taken together, as described above, our results reveal that all the investigated positions had differential effects with regard to glycine- and Cs^+^-dependent current responses in comparison with the non-mutated GlyR α3L^185L^. In particular, mutation of positions D97 and Y161 had a detrimental effect on GlyR α3L^185L^ activation, with the exception of Y161A retaining some Cs^+^-evoked current responses at concentrations above 50 mM. Furthermore, alanine substitutions at positions D91, E110, and D114 decreased apparent glycine- and Cs^+^-affinities (EC_50_) and cooperativity (Hill slope), with the exception of the E110A mutant not affecting Cs^+^-dependent response parameters. Lysine substitutions at these positions had no effect with the exception of D91K variants also showing decreased apparent glycine- and Cs^+^-affinities (EC_50_) and cooperativity (Hill slope). Notably, and in contrast to our expectations, none of the mutations selectively abolished Cs^+^-evoked responses while leaving glycine-dependent ones unaffected.

Alterations of mutant current responses could also result from deficits in the cell surface expression of the different GlyR mutants. To address this possibility, we performed a quantitative analysis of the surface expression for the different GlyR mutants, which were stained using the N-terminally inserted HA-epitope tag and a validated life cell staining protocol (Harnau et al., 2025). A priori, this hypothesis would mainly concern receptor mutants that displayed lack of both, glycine- and Cs^+^-evoked current responses. These are D97A, D97K, and Y161K. Figure 3A shows representative confocal images of HE293T cells expressing the non-mutated reference clone GlyR α3L^185L^, EGFP only, and the diverse GlyR mutants. All GlyRs were expressed at the cell surface, but the quantification of absolute fluorescence and EGFP-normalized values revealed significant differences (Figures 3B,C, Table 3, *p < 0.05, **p < 0.01). Nevertheless, D97A/K mutants were detectable at the cell surface (Figures 3A–C, Table 3), while they were non-functional mutants with regard to agonist-dependent receptor activation – ruling out the aforementioned hypothesis in these cases. Y161A/K mutants showed equally well reduced surface expression levels (Figures 3A,B, Table 3, **p < 0.05), but the Y161A mutant retained significant Cs^+^-dependent current responses (Figures 2M–O, Table 1), again suggesting that deficits in surface expression were not responsible for the observed effects of A/K-substitutions on current responses in the GlyR mutants. Thus, changes in receptor surface expression do not seem to be a reason for the observed effects of the mutations on receptor activation.

**Figure 3:**
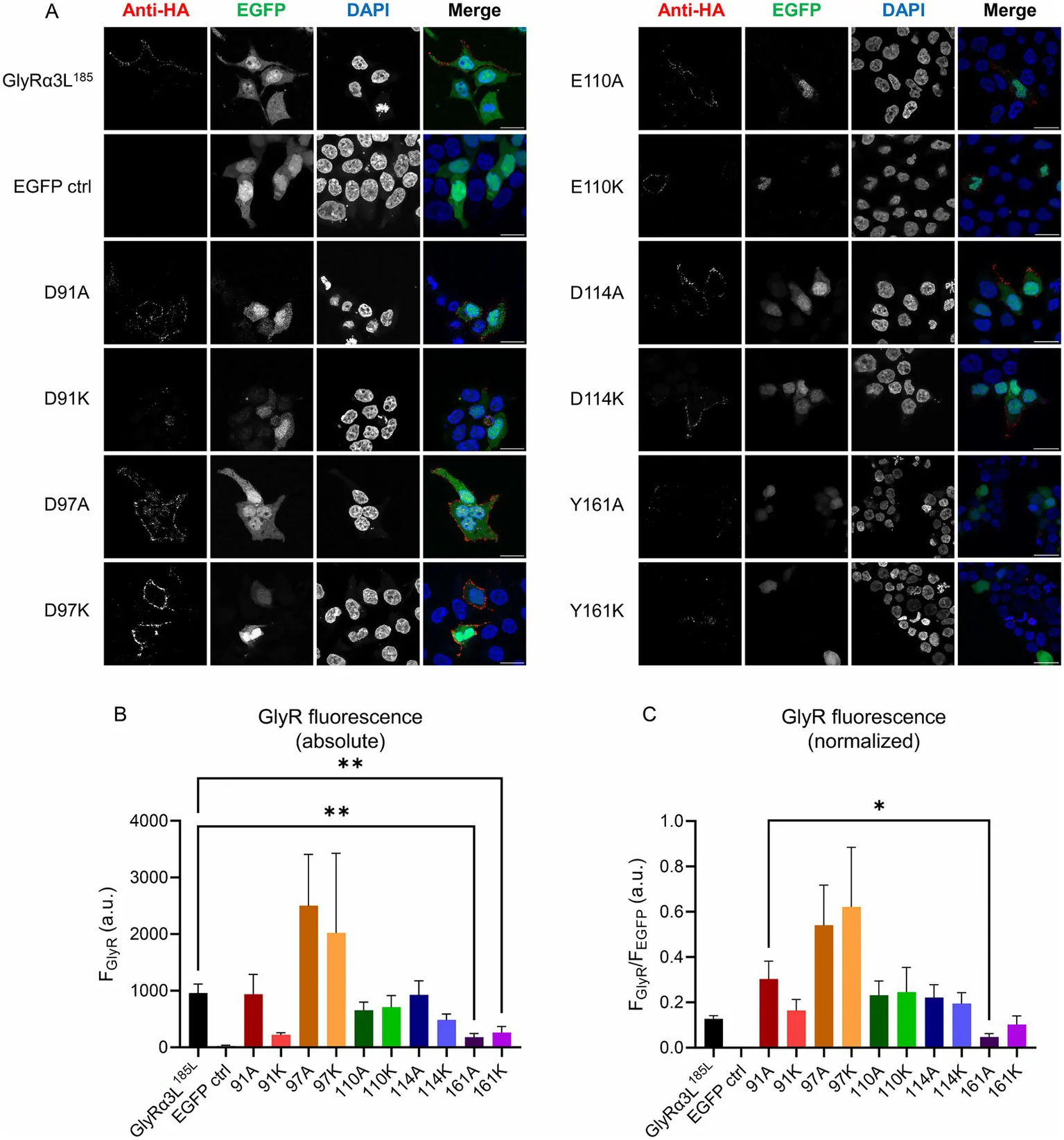
Analysis of surface expression of GlyR mutants. **(A)** Representative confocal images of surface-stained GlyRs in HEK293T cells. The non-mutated GlyR α3L^185L^ served as positive reference control, while cells expressing solely EGFP served as negative controls with regard to HA-dependent life cell staining of the GlyR mutants D91A/K, D97A/K, E110A/K, D114A/K, and Y161A/K. Scale bars are 20 µm. **(B,C)** Quantification of the absolute **(B)** and EGFP-normalized GlyR surface immunoreactivity **(C)** of HEK293T cells expressing either GlyR α3L^185L^ (*n* = 19), EGFP (*n* = 10), D91A (*n* = 15), D91K (*n* = 11), D97A (*n* = 16), D97K (*n* = 14), E110A (*n* = 15), E110K (*n* = 15), D114A (*n* = 16), D114K (*n* = 15), Y161A (*n* = 15) and Y161K (*n* = 15). Data are presented as mean ± SEM (*p < 0.05, **p < 0.01).

**Table 3:** Surface localization of non-mutated and mutated GlyR α3L^185L^ in HEK293T cells (in absolute fluorescence per cell and EGFP-normalized).

	F/cell (a.u.)	(F/cell) / (EGFP/cell)
GlyR α3L^185L^ (*n* = 19)	958.48 ± 161.37 (SD = 703.38)	0.13 ± 0.01 (SD =0.06)
EGFP ctrl (*n* = 10)	29.24 ± 7.92 (SD = 25.03)	0.00 ± 0.00 (SD = 0.00)
D91A (*n* = 15)	937.19 ± 349.88 (SD =1355.09)	0.30 ± 0.08 (SD =0.31)
D91K (*n* = 11)	223.23 ± 34.27 (SD =113.66)	0.16 ± 0.05 (SD =0.16)
D97A (*n* = 16)	2502.11 ± 906.51 (SD = 3626.05)	0.54 ± 0.18 (SD = 0.71)
D97K (*n* = 14)	2023.00 ± 1406.79 (SD = 5263.73)	0.62 ± 0.26 (SD = 0.98)
E110A (*n* = 15)	656.32 ± 142.95 (SD = 553.63)	0.23 ± 0.06 (SD = 0.25)
E110K (*n* = 15)	709.01 ± 204.47 (SD = 791.92)	0.25 ± 0.11 (SD = 0.42)
D114A (*n* = 16)	928.97 ± 244.57 (SD =978.29)	0.22 ± 0.06 (SD =0.23)
D114K (*n* = 15)	486.19 ± 101.11 (SD =391.61)	0.20 ± 0.05 (SD =0.19)
Y161A (*n* = 15)	178.14 ± 65.21 (SD = 252.56)	0.05 ± 0.02 (SD = 0.06)
Y161K (*n* = 15)	260.52 ± 107.12 (SD = 107.12)	0.10 ± 0.04 (SD = 0.15)

Data are listed as mean ± SEM (and SD).

## Discussion

Aim of this study was to screen amino acid positions in or near the orthosteric glycine binding site of the homomeric GlyR α3L^185L^ with regard to their relevance for Cs^+^-dependent receptor activation, as the positions D91, D97, E110, D114, and Y161 were predicted in a recent study (Fricke et al., 2023). While the results do not identify a critical position selectively involved in Cs^+^-dependent receptor activation, they show that many of these positions are relevant for glycine- and Cs^+^-dependent receptor activation.

Alanine and lysine substitutions at these positions identify D97 and Y161 as critical positions for GlyR α3L^185L^ activation in general as they produced non-functional mutants with regard to response to application of the agonists, with the exception of Y161A retaining Cs^+^-evoked receptor current responses. D91A/K mutants reduced peak current amplitudes and decreased the apparent affinities in response to both glycine and Cs^+^. D114A displayed significantly reduced currents only at low Cs^+^ concentrations and showed a reduction of the apparent affinities for glycine and Cs^+^. Finally, alanine and leucine substitutions at the position E110 selectively affected glycine-evoked current responses, with E110A also showing reduced apparent affinity for glycine. Thus, none of these mutations selectively abolished Cs^+^-dependent receptor activation.

Analysis of the cell surface expression of the GlyR mutants revealed some reduction particularly concerning the Y161 mutants. However, D97 and other mutants with reduced peak current responses to Cs^+^ and/or glycine were not compromised with regard to receptor surface expression. Thus, the observed effects were not due to reduced receptor surface expression, but rather involve structural alterations resulting in changes of receptor activation.

The extracellular ligand binding domain of so-called Cys-loop receptors including GlyRs consists of different β-sheets interconnecting the so-called loops (Tang and Lummis, 2018). In particular, D91 and D97 are located between β-sheets 3 and 4 preceding the A-loop, and E110 and D114 are between β-sheets 5 and 5’, with E110 and D114 immediately following the A-loop and preceding the E-loop. Y161 is between β-sheets 7 and 8 directly in the B-loop in the orthosteric glycine binding site. How do all these structural features interconnect ligand binding and channel gating? It is known that agonist binding triggers local electrostatic shifts alter the charge balance and break resting-state salt-bridges (such as those involving loops A and B; Cederholm et al., 2009; Chang et al., 2009). A conformational wave follows, involving a twisting of the β-sheets around the Cys-loop axis. This triggers the Cys-loop and surrounding loops to shift positions and generate mechanical and electrostatic pulling forces that reach the membrane-spanning domain via the linker between transmembrane domains 2 and 3, finally opening the central ion pore (Cederholm et al., 2009; Chang et al., 2009).

In a recent study (Harnau et al., 2025), we had already investigated amino acids D141, E192, and D194 in the Cys-loop (D141) or on β-sheet 9 (E192, D194), which were also predicted previously (Fricke et al., 2023), and identified D194 as a relevant position for both glycine- and Cs^+^-dependent receptor activation. Thus, our current study also gets loops A and B involved in the structural framework for GlyR activation. Together with earlier results (Legendre et al., 2009; Harnau et al., 2025), showing the relevance of the F-loop and β-sheet 9, our results suggest that electrostatic changes in these critical domains affect receptor activation, either directly through orthosteric domain mechanisms or remotely via the region of the F-loop. Cs^+^ could also interact with glycine and convert the neurotransmitter glycine from neutral to its zwitterionic form, thereby changing electrostatic properties (Wang et al., 2025). However, such an effect can be ruled out because Cs^+^-dependent current responses were elicited in the absence of glycine in the Cs^+^-containing application solutions.

Besides homomeric GlyRs with only α-subunits, the GlyR β-subunit can co-assemble with α-subunits into heteromeric α/β-GlyRs, which influences ligand binding properties of synaptic GlyRs (Grudzinska et al., 2005) and current-voltage relationships of glycine-evoked currents (Raltschev et al., 2016). As we studied here and in previous studies (Harnau et al., 2025) only homomeric GlyR α3L^185L^ and homomeric α3K/L- and RNA-edited α3K/L^185L^-GlyRs as well as α2A/B- and RNA-edited α2A/B^192L^-GlyRs (Fricke et al., 2023), the possibility that the GlyR β-subunit contributes to Cs^+^-dependent current responses remains to be explored and is a limitation of this and our previous studies.

Another monovalent cation, NH_4_^+^, was also recently discovered to be an agonist of RNA-edited or non-RNA-edited α2- and α3-GlyRs (Kankowski et al., 2018), whereas Li^+^, Na^+^, and K^+^ were not able to induce GlyR-dependent transmembrane currents (Fricke et al., 2023). In fact, NH_4_^+^ and Cs^+^ are monovalent cations with similar ionic radii, which leads to comparable behaviors in ion-exchange processes. Thus, our results suggest that any monovalent cation with a similar ionic radius would also be able to activate GlyRs, and they also suggest that Cs^+^-dependent GlyR activation involves an electrostatic structural framework with possibly many Cs^+^-binding sites in the extracellular GlyR ligand binding domain. In contrast to glycine-dependent receptor activation involving distinguished amino acid positions, Cs^+^ could hence exert electrostatic “pressure” on the GlyR protein structure and its transition to an open channel conformation. This hypothesis is supported by the fact that no mutant was identified that responded to glycine while lacking Cs^+^-evoked receptor activation. Alternatively, we might just not yet have uncovered the amino acid positions or combinations of them that could be selectively involved in Cs^+^-dependent GlyR activation.

## Data Availability

The original contributions presented in the study are included in the article/Supplementary material, further inquiries can be directed to the corresponding author.
